# Gut microbiota manipulation during the prepubertal period shapes behavioral abnormalities in a mouse neurodevelopmental disorder model

**DOI:** 10.1038/s41598-020-61635-6

**Published:** 2020-03-13

**Authors:** Justin M. Saunders, José L. Moreno, Daisuke Ibi, Masoumeh Sikaroodi, Dae Joong Kang, Raquel Muñoz-Moreno, Swati S. Dalmet, Adolfo García-Sastre, Patrick M. Gillevet, Mikhail G. Dozmorov, Jasmohan S. Bajaj, Javier González-Maeso

**Affiliations:** 1 Department of Physiology and Biophysics, Virginia Commonwealth University School of Medicine, Richmond, VA 23298 USA; 2 Division of Gastroenterology, Hepatology and Nutrition, Virginia Commonwealth University and McGuire VA Medical Center, Richmond, VA 23298 USA; 3 Department of Biostatistics, Virginia Commonwealth University School of Medicine, Richmond, VA 23298 USA; 4 Center for Microbiome Analysis, George Mason University, Manassas, VA 20110 USA; 5 Department of Microbiology and Global Health & Emerging Pathogens Institute, Icahn School of Medicine at Mount Sinai, New York, NY 10029 USA; 6 Department of Medicine – Division of Infectious Diseases, Icahn School of Medicine at Mount Sinai, New York, NY 10029 USA; 7 The Tisch Cancer Institute, Icahn School of Medicine at Mount Sinai, New York, NY 10029 USA; 8 Present Address: VIVEbiotech S.L., E-20009, Donostia/San Sebastián, Spain; 9 Present Address: Department of Chemical Pharmacology, Meijo University, Nagoya, 468–8503 Japan

**Keywords:** Disease model, Microbiome, Schizophrenia

## Abstract

Previous studies demonstrate an association between activation of the maternal immune system during pregnancy and increased risk of neurodevelopmental psychiatric conditions, such as schizophrenia and autism, in the offspring. Relatively recent findings also suggest that the gut microbiota plays an important role in shaping brain development and behavior. Here we show that maternal immune activation (MIA) accomplished by infection with a mouse-adapted influenza virus during pregnancy induced up-regulation of frontal cortex serotonin 5-HT_2A_ receptor (5-HT_2A_R) density in the adult offspring, a phenotype previously observed in postmortem frontal cortex of schizophrenic subjects. 5-HT_2A_R agonist-induced head-twitch behavior was also augmented in this preclinical mouse model. Using the novel object recognition (NOR) test to evaluate cognitive performance, we demonstrate that MIA induced NOR deficits in adult offspring. Oral antibiotic treatment of prepubertal mice prevented this cognitive impairment, but not increased frontal cortex 5-HT_2A_R density or psychedelic-induced head-twitch behavior in adult MIA offspring. Additionally, gut microbiota transplantation from MIA mice produced behavioral deficits in antibiotic-treated mock mice. Adult MIA offspring displayed altered gut microbiota, and relative abundance of specific components of the gut microbiota, including *Ruminococcaceae*, correlated with frontal cortex 5-HT_2A_R density. Together, these findings provide a better understanding of basic mechanisms by which prenatal insults impact offspring brain function, and suggest gut-brain axis manipulation as a potential therapeutic approach for neurodevelopmental psychiatric conditions.

## Introduction

Neurodevelopmental psychiatric disorders, including schizophrenia^1^ and autism^2^, are severe and usually cause life-long disability. Epidemiological studies have indicated that environmental insults during pregnancy, particularly infection and severe adverse life events, increase the risk of certain psychiatric disorders in offspring. Thus, it has been reported that maternal infection with agents including viruses (influenza and rubella)^3,4^, bacteria (bronchopneumonia)^5^ and protozoa (*Toxoplasma gondii*)^6^ contribute to the etiologies of neuropsychiatric disorders such as schizophrenia and autism. Converging lines of evidence from humans^7^ and rodent models^8^ suggest a link between maternal immune activation (MIA) during pregnancy and abnormalities in offspring brain structure and function. Indeed, rodent^9^ and non-human primate^10^ animal models have demonstrated a causal relationship between MIA and neuropathological abnormalities.

There is growing awareness that crosstalk between intestinal bacteria and the CNS is critically important for maintaining homeostasis and plasticity^11,12^. Three clinical studies demonstrate significant differences in composition of the oropharyngeal^13,14^ and gut^15^ microbiome between schizophrenia patients and controls, with similarly altered gut bacterial profiles found in children with autism^16,17^. Recent preclinical findings also suggest that MIA in mice produces dysbiosis of the intestinal microbiota^18–20^ and abnormal behavioral phenotypes in offspring^18,21^. This accumulating evidence supports the existence of altered gut-brain physiological pathways in both MIA models and neurodevelopmental psychiatric conditions. In light of this, antibiotic-induced manipulation of the gut microbiota may serve as a tool to evaluate the contribution of microbiome alterations to the onset of cognitive deficits in mouse prenatal insult models. Other groups have recently reported that prepubertal administration of minocycline – a tetracycline antibiotic also used to inhibit microglial activation – attenuates abnormal behavior in MIA offspring^22,23^. Nevertheless, molecular mechanisms within the gut-brain axis that are involved in these phenotypes remain largely unclear.

**Figure 1:**
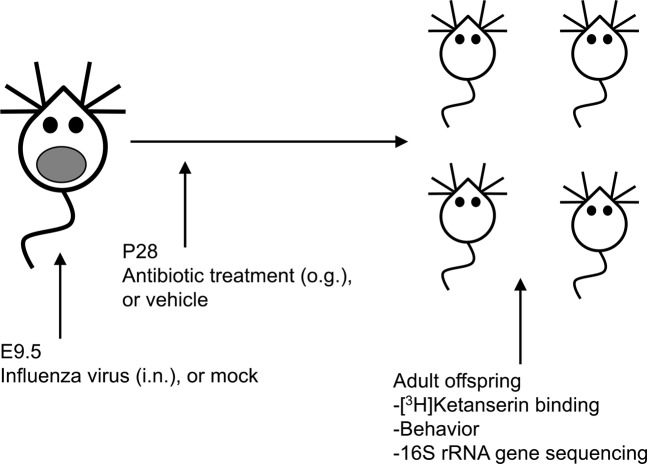
Schematic representation of a prenatal MIA model. Timed pregnant mice (E9.5) were inoculated (i.n.) with influenza A/WSN/33 (H1N1) virus (5 × 10^3^ pfu), or mock (PBS). After this manipulation, prepubertal (28 d) mice born to influenza virus-infected mothers or mock mothers received a single dose of streptomycin (20 mg), or vehicle by oral gavage. Biochemical, behavioral and 16S rRNA gene sequencing assays were carried out in adult mice.

The serotonin 5-HT_2A_ receptor (5-HT_2A_R) is a family A G protein-coupled receptor (GPCR) involved in mechanisms related to cognition, perception and sensorimotor gating^24,25^. We previously showed up-regulation of the 5-HT_2A_R in postmortem frontal cortex of untreated schizophrenic subjects^26–28^ – a pattern of expression that could predispose to certain schizophrenia symptoms. Remarkably, in three independent mouse models of prenatal insults: influenza virus infection^29^, variable and unpredictable stress^8^, and immune activation with poly-(I:C)^8^, we found up-regulation of cortical 5-HT_2A_R expression in association with behavioral changes indicative of deficits in perception and cognition^8,29^, findings that others have validated using a variety of prenatal insult models^30–32^. Using maternal infection with a mouse adapted influenza A/WSN/33 (H1N1) virus as a preclinical MIA model (Fig. 1), here we asked whether manipulation of the gut-brain axis could serve as a novel approach to reduce transition to MIA-induced phenotypes, including dysregulation of frontal cortex 5-HT_2A_R density and altered performance in behavioral models of psychosis and cognition.

## Results

### Prepubertal gut microbiota manipulation does not affect MIA-induced psychosis-related phenotypes later in life

**Figure 2:**
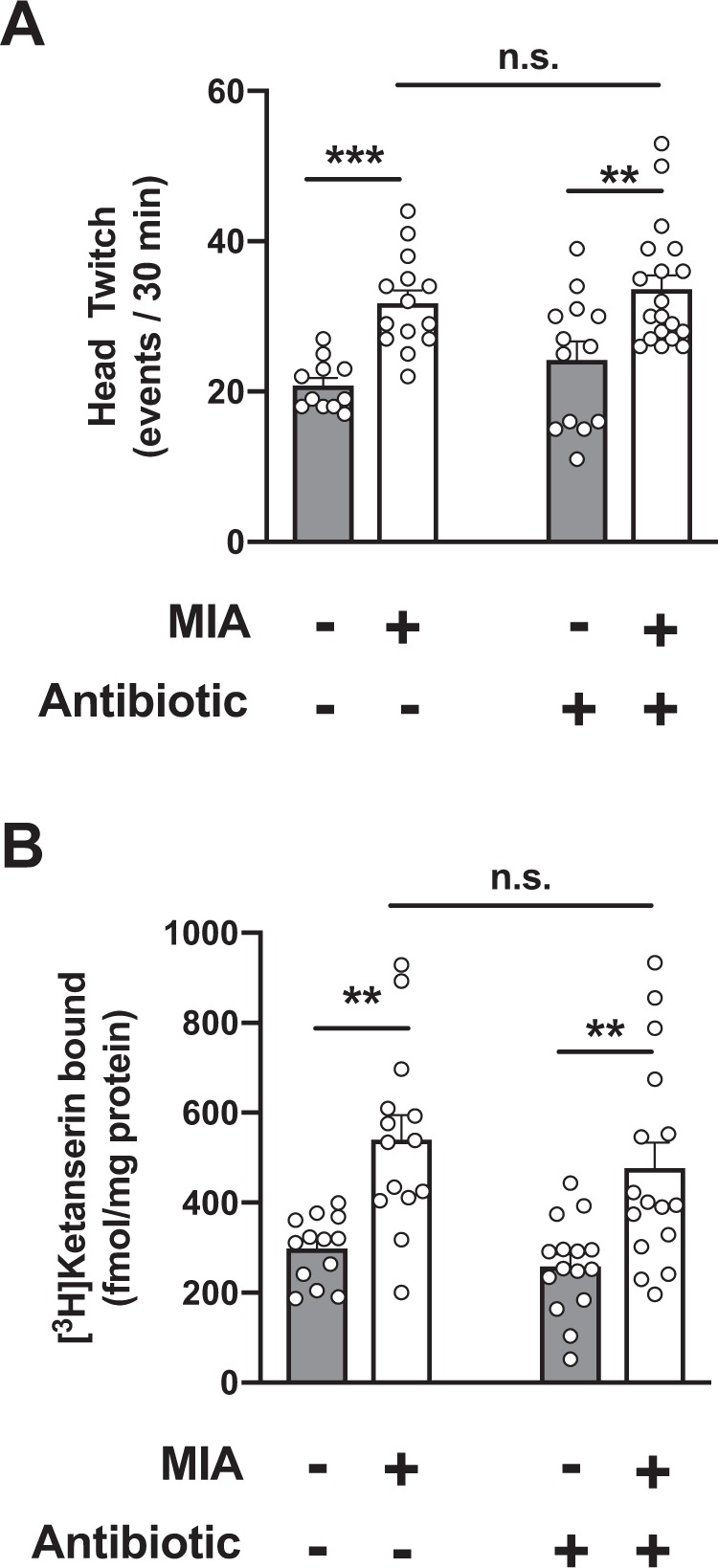
Juvenile mice born to influenza virus-infected mothers (MIA) and controls (mock) received a single dose of antibiotic or vehicle via oral gavage. Head-twitch behavior (**A**) and [^3^H]ketanserin binding assays (**B)** were performed in adult mice. (**A**) Effect of injection with the psychedelic 5-HT_2A_R agonist DOI (0.5 mg/kg), or vehicle, on head-twitch behavior (n = 11 – 19). (**B**) Radioligand binding with the 5-HT_2A_R antagonist [^3^H]ketanserin (10 nM) in mouse frontal cortex membrane preparations (n = 13 – 16). Two-way ANOVA (**A**) F[1,53] = 27.89, *p* < 0.001; (**B**) F[1,54] = 27.07, *p* < 0.001). Bonferroni’s post hoc test (*p < 0.05, **p < 0.01, ***p < 0.001, n.s., not significant). Data show mean ± s.e.m.

Serotonergic psychedelics, such as lysergic acid diethylamide (LSD) and psilocin, induce in healthy volunteers alterations in perception and sensory processing that show certain similarities to those observed in schizophrenia patients suffering from psychotic episodes^33,34^. Our previous observations clearly demonstrated that head-twitch behavior represents a mouse behavioral proxy of human psychedelic drug action^35,36^. Using the psychedelic 5-HT_2A_R agonist 1-(2,5-dimethoxy-4-iodophenyl)-2-aminopropane (DOI) as a pharmacological tool, we already reported that DOI-induced head-twitch behavior was augmented in adult mice born to influenza virus-infected mothers, as compared to control adult mice born to mock-infected mothers^29^. Consequently, as expected, our findings here show that both head-twitch behavior induced by DOI (0.5 mg/kg) (Fig. 2A) and frontal cortex 5-HT_2A_R density as measured by radioligand binding assays with the 5-HT_2A_R antagonist [^3^H]ketanserin (Fig. 2B) were significantly increased in adult MIA-mice, as compared to adult mice born to mock-infected mothers.

Symptoms of schizophrenia generally first appear during late adolescence or early adulthood^1^. Unfortunately, results of several clinical studies clearly show that the available antipsychotic medications do not delay or prevent conversion to psychosis or memory deficits in young subjects with initial prodromal symptoms^37,38^. This underlines the need for new therapeutic strategies to prevent the onset of these psychiatric conditions. Prebiotics are dietary non-digestible fibers that beneficially affect the host’s health mostly by selectively promoting growth and activity of some genera of microorganisms in the colon, generally *Lactobacilli* or *Bifidobacteria*^39^. Previous findings suggest that administration via the drinking water of a specific mixture of non-digestible galacto-oligosaccharides for three weeks prevents the effect of a single injection of lipopolysaccharide on up-regulation of 5-HT_2A_R immunoreactivity in the mouse frontal cortex^40^. Based on these findings, together with our previous observations suggesting that up-regulation of frontal cortex 5-HT_2A_R density is not observed in prepubertal MIA mice^8,29^, here we tested the extent to which microbiota manipulation during a prepubertal period normalizes MIA-induced alterations in 5-HT_2A_R expression and 5-HT_2A_R-dependent behaviors. To do so, mice born to influenza virus-infected mothers during pregnancy or controls received a single dose of antibiotic treatment via oral gavage, or vehicle during the prepubertal period (P28). After this manipulation, mice were housed with normal food and water until they became adult animals (Fig. 1). Increases in head-twitch behavior induced by the psychedelic 5-HT_2A_R agonist DOI (Fig. 2A) and density of frontal cortex 5-HT_2A_R when assayed by [^3^H]ketanserin binding (Fig. 2B) were observed in adult MIA offspring relative to mock offspring regardless of antibiotic treatment at P28.

### Prepubertal gut microbiota manipulation prevents MIA-induced deficits in recognition memory

**Figure 3:**
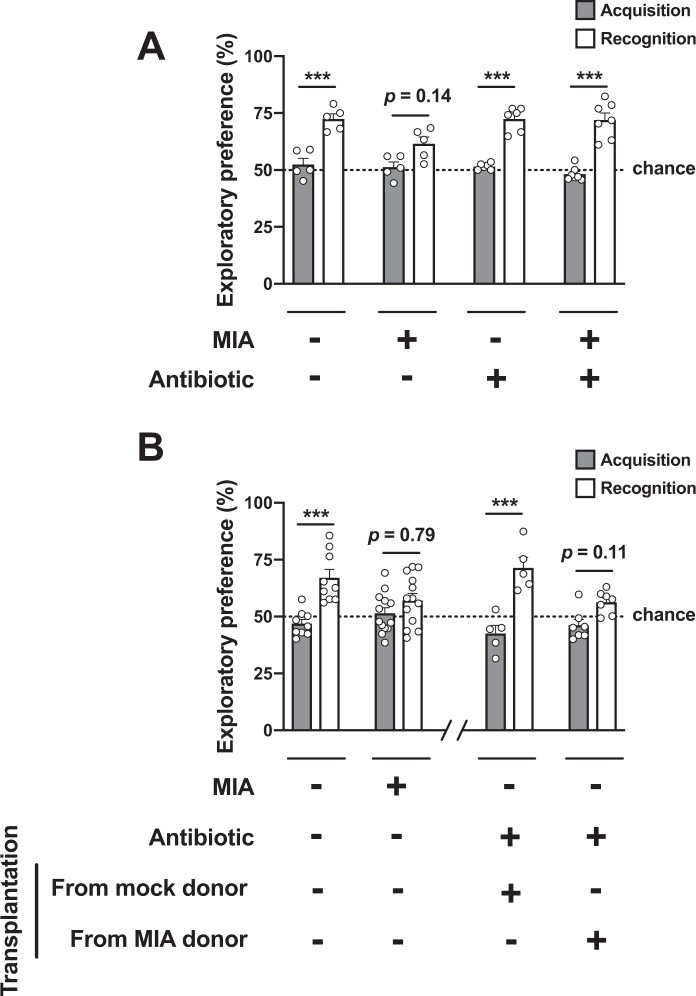
(**A**) Juvenile mice born to influenza virus-infected mothers and controls received a single dose of antibiotic via oral gavage, or vehicle. This paradigm of gut microbiota manipulation prevents MIA-induced cognitive deficits in adult mice. (**B**) Offspring mice born to influenza virus-infected mothers and controls were allowed to reach adulthood (*left side of the panel*). Juvenile mice born to control mothers received a single dose of antibiotic via oral gavage, or vehicle. After this manipulation, juvenile mice received fecal microbiota transplantation from adult MIA or mock mice (*right side of the panel*). Novel object recognition was tested in adult animals (n = 5 – 7 for (**A**) n = 5 – 13 for (**B)**). Two-way ANOVA (**A**) F[1,38] = 135.6, *p* < 0.001, see also Table S1; (**B**) *(left side of the panel)*, F[1,40] = 19.76, *p* < 0.001; (**B**) *(right side of the panel)*, F[1,20] = 40.14, *p* < 0.001). Bonferroni’s post hoc test (*p < 0.05, **p < 0.01, ***p < 0.001, n.s., not significant). Data show mean ± s.e.m.

Previous findings including ours suggest that certain models of prenatal insults negatively affect offspring’s cognitive capabilities^8,18,21^. To test whether prepubertal gut microbiota manipulation modulates MIA-induced cognitive deficits, we evaluated the effect of antibiotic administration via oral gavage at P28 on a novel-object recognition test in adult mice as a measure of cognitive performance. Our results show that novel-object recognition performance was significantly disrupted in adult MIA offspring as compared to adult mock offspring mice when both groups were gavaged with vehicle at P28 (Fig. 3A and Table S1). Importantly, our results also show that prepubertal gut microbiota modulation at P28 prevents the effect of prenatal MIA on adult offspring’s recognition memory performance (Fig. 3A and Table S1).

### Microbiota transplantation from MIA mice negatively affects recognition memory

In order to investigate whether the gut microbiota was responsible for triggering cognitive deficits in MIA mice, we transplanted the cecal microbiota from adult MIA mice into young mice born to mock-infected mothers, and tested cognitive capabilities later in life. As before (see Fig. 3A), adult MIA offspring mice show deficits in recognition memory as compared to adult mice born to mock-infected mothers during pregnancy (Fig. 3B). Importantly, adult mice born to mock-infected mothers and transplanted at a prepubertal period with microbiota from adult MIA mice showed behavioral deficits in recognition memory performance (Fig. 3B). This negative effect of gut microbiota manipulation, however, was not observed in adult mice born to mock-infected mothers and transplanted at a prepubertal period with gut microbiota from adult control mice (Fig. 3B).

### Influenza virus infection during pregnancy alters adult offspring’s gut microbiota composition, which is rebalanced by juvenile antibiotic treatment

**Figure 4:**
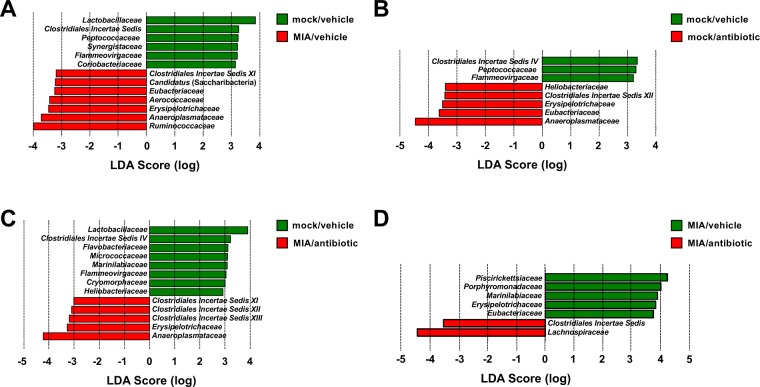
Effect of prepubertal antibiotic administration on MIA-induced dysbiosis of the gut microbiota. Juvenile (P28) mice born to influenza virus-infected (MIA) mothers, or mock mothers, received a single dose of antibiotic, or vehicle, via oral gavage at P28. Cecal samples were collected from adult mice and analyzed by linear discriminant analysis effect size (LEfSe). Data are presented in a histogram with Linear Discriminant Analysis (LDA) between groups (n = 9 – 13). (**A**) mock (juvenile vehicle) vs. MIA (juvenile vehicle). (**B**) mock (juvenile vehicle) vs. mock (juvenile antibiotic). (**C**) mock (juvenile vehicle) vs. MIA (juvenile antibiotic). (**D**) MIA (juvenile vehicle) vs. MIA (juvenile antibiotic).

Abnormalities related to the gut microbiota composition have previously been reported in MIA and maternal gestational stress models^18,20^. To determine the impact of maternal viral infection during the second week of pregnancy on the murine gut microbial community, we surveyed the cecal bacterial composition by 16S rRNA gene sequencing of samples isolated from adult mice born to mothers intranasally exposed to influenza virus, or mock. We used the LEfSe algorithm to define the potential differential bacterial patterns in cecum samples^41^. Our data show that a total of seven bacterial families were overrepresented in MIA mice – these included *Ruminococcaceae*, *Porphyromonadaceae, Aerococcaceae*, and *Erysipelotrichaceae* (Fig. 4). Similar findings have been observed in the feces of subjects with psychiatric conditions, including schizophrenia, autism and major depressive disorder^14,15,42–44^. *Candidatus* (saccharibacteria), an obligate epibiont^45^, was also more abundant in MIA offspring (Fig. 4). Conversely, adult offspring mice born to mock-infected mothers during pregnancy possessed a gut microbiota enriched in a total of six bacterial families. These included members of the usually beneficial taxa^11,46–49^ belonging to *Lactobacillaceae*, *Peptococcaceae*, and *Coriobacteriaceae* (Fig. 4). This concept of dysbiosis of the adult gut microbiota induced by maternal infection with a mouse-adapted influenza virus was confirmed by comparison of RDP11 classifier values (Table 1 and Fig. 4).

**Table 1:** Relative abundances of bacterial taxa in adult mice born to influenza virus-infected mothers (MIA), or mock, and prepubertally (P28) treated with antibiotics, or vehicle.

Taxa (family level)	mock – vehicle	MIA – vehicle	mock – antibiotic	MIA – antibiotic	*p* value
*Erysipelotrichaceae*	0.0022 ± 0.00031	0.0067 ± 0.00069	0.0056 ± 0.0014	0.0046 ± 0.0005	0.0005
*Eubacteriaceae*	0.00014 ± 0.0001	0.0015 ± 0.00038	0.0022 ± 0.00056	0.0004 ± 0.00022	0.0007
*Porphyromonadaceae*	0.053 ± 0.0052	0.065 ± 0.0069	0.045 ± 0.0068	0.044 ± 0.0028	0.0042
*Carnobacteriaceae*	0.00016 ± 0.0002	N.D.	0.0011 ± 0.00044	N.D.	0.0047
*Marinilabiaceae*	0.0020 ± 0.0006	0.0044 ± 0.0010	0.0017 ± 0.00035	0.0005 ± 0.00022	0.0073
*Coriobacteriaceae*	0.0016 ± 0.00025	0.00058 ± 0.0002	0.0013 ± 0.00019	0.0011 ± 0.00023	0.014
*Peptococcaceae*	0.0042 ± 0.00070	0.0023 ± 0.00031	0.0019 ± 0.00048	0.0042 ± 0.00066	0.014
*Clostridiales Incertae Sedis IV*	0.0039 ± 0.00093	0.0017 ± 0.00037	0.00071 ± 0.00037	0.0015 ± 0.00038	0.015
*Flavobacteriaceae*	0.0021 ± 0.00014	0.0021 ± 0.00014	0.0016 ± 0.00024	0.0015 ± 0.00016	0.018
*Heliobacteriaceae*	0.0018 ± 0.00014	0.0018 ± 0.00022	0.0026 ± 0.00048	0.0014 ± 0.00019	0.022
*Lactobacillaceae*	0.024 ± 0.0057	0.0094 ± 0.0013	0.0129± 0.0025	0.0086 ± 0.0019	0.023
*Clostridiales Incertae Sedis XII*	0.0016 ± 0.00011	0.0016± 0.00020	0.0026 ± 0.00030	0.0021 ± 0.00013	0.024
*Clostridiales Incertae Sedis XI*	0.00054 ± 0.00021	0.0020 ± 0.00054	0.00089 ± 0.00023	0.0021 ± 0.00051	0.025
*Anaeroplasmataceae*	N.D.	0.010 ± 0.0035	0.055 ± 0.018	0.030 ± 0.015	0.028
*Aerococcaceae*	N.D.	0.00069 ± 0.0002	0.00016 ± 0.0001	0.00013 ± 0.0001	0.033
*Candidatus* (saccharibacteria)	0.00062 ± 0.00034	0.0024 ± 0.00056	0.00055 ± 0.00029	0.0029 ± 0.00084	0.035
*Clostridiales Incertae Sedis*	0.0043 ± 0.0010	0.0018 ± 0.00013	0.0024 ± 0.00047	0.0035 ± 0.00058	0.039
*Flammeovirgaceae*	0.0010 ± 0.00034	0.00015 ± 0.0001	0.00013 ± 0.0001	N.D.	0.042
*Piscirickettsiaceae*	0.00029 ± 0.00019	0.00085 ± 0.0003	0.055 ± 0.018	0.00014 ± 0.0001	0.052
*Ruminococcaceae*	0.10 ± 0.004	0.12 ± 0.004	0.11 ± 0.005	0.10 ± 0.005	0.07
*Micrococcaceae*	0.00038 ± 0.00019	0.00009 ± 0.003	0.00012 ± 0.00036	N.D.	0.11
*Cryomorphaceae*	0.0010 ± 0.00020	0.00079 ± 0.0002	0.00096 ± 0.00018	0.00056 ± 0.0001	0.20
*Synergistaceae*	0.0024 ± 0.00014	0.0015 ± 0.00028	0.0018 ± 0.00049	0.0019 ± 0.00037	0.23

Taxa are listed in order of significance (Kruskal-Wallis test). All values are presented as mean ± s.e.m. N.D., not detected.

**Figure 5:**
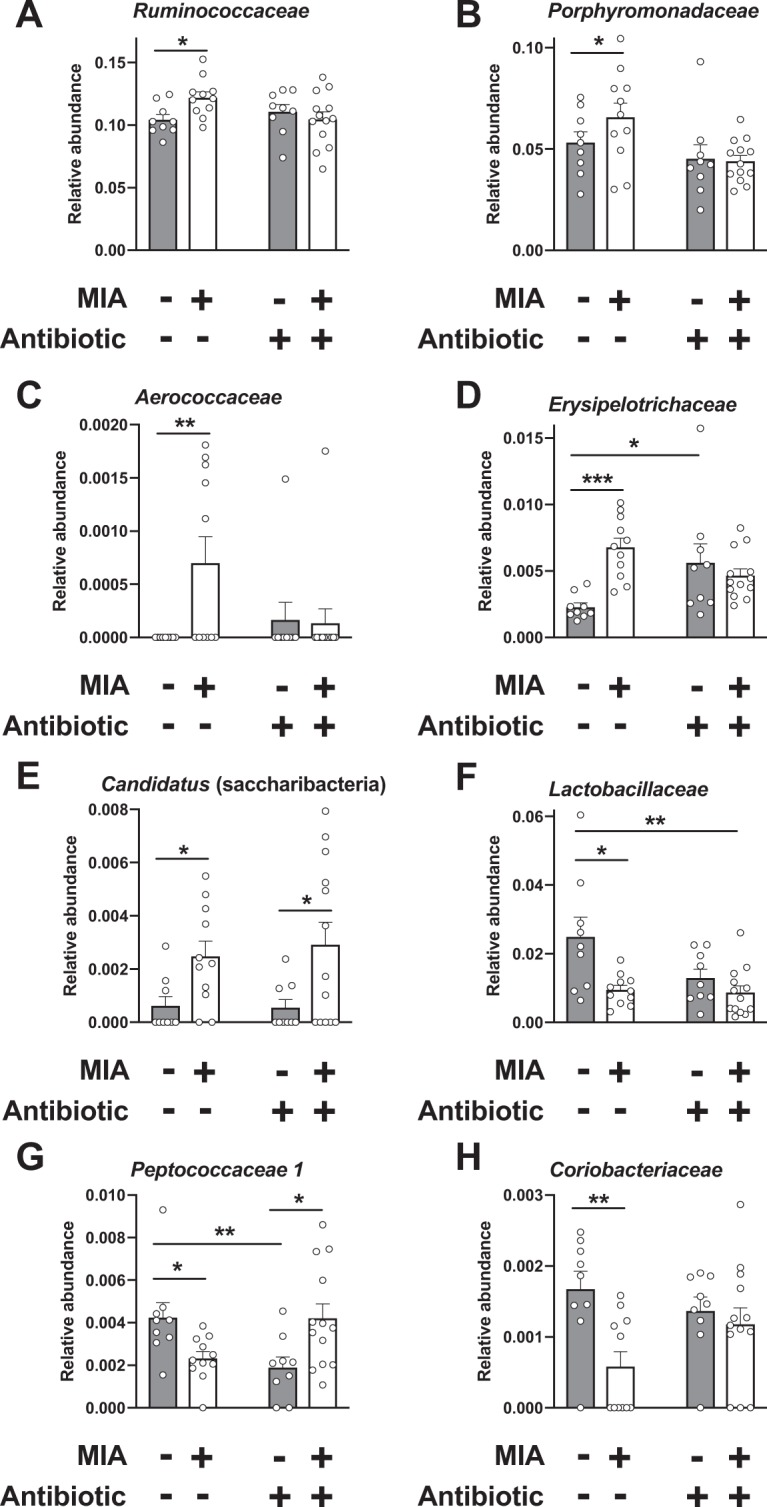
Effect of antibiotic administration or vehicle via oral gavage at P28 on relative abundances at the bacterial family level in mice born to influenza virus-infected (MIA) mothers, or mock mothers (n = 9 – 13). Cecal samples were collected from adult offspring mice. Kruskal-Wallis (see Table 1) followed by uncorrected Dunn’s post hoc test (*p < 0.05, **p < 0.01, ***p < 0.001, n.s., not significant). Data show mean ± s.e.m.

We next tested the extent to which treatment of young mice with antibiotics that are not systemically absorbed from the intestine remodels MIA-induced alterations in gut microbiota composition later in life. As above, MIA and mock mice received at P28 a single dose of streptomycin via oral gavage, or vehicle, after which they were allowed access to normal food and water until adulthood. Our data show a relatively low number of alterations in gut microbiota composition of adult offspring born to mock-infected mothers and injected with antibiotic at a prepubertal age, as compared to adult offspring born to mock-infected mothers and injected with vehicle at a prepubertal age (Fig. 4). Examples of these changes induced by prepubertal antibiotic treatment included reduction in *Peptococcaceae*, as well as augmentation of *Erysipelotrichaceae* (Table 1, and Figs. 4 and 5).

Our data also suggest the notably novel concept that dysbiosis of the gut microbiota in adult mice born to influenza virus-infected mothers can be partially rebalanced by prepubertal gut microbiota manipulation via antibiotic administration. Thus, potentially pathogenic taxa belonging to *Ruminococcaceae*, *Porphyromonadaceae* and *Aerococcaceae* were not augmented in adult MIA offspring prepubertally treated with antibiotics (Table 1, and Figs. 4 and 5). We also show that the known beneficial family *Peptococcaceae*, which, as described above, was reduced in adult MIA offspring injected with vehicle at P28 as compared to adult mock offspring injected with vehicle at P28, exhibited an interesting higher relative abundance in adult MIA offspring injected with streptomycin at P28 as compared to adult mock offspring injected with streptomycin at P28 (Table 1, and Figs. 4 and 5). Other alterations induced by prenatal MIA, such as augmentation of *Candidatus* (saccharibacteria) and *Clostridiales Incertae Sedis XI*, were unaffected by prepubertal antibiotic treatment (Table 1, and Figs. 4 and 5). Principal coordinate analysis (PCO) shows absence of overall gut microbiota community change in adult MIA offspring injected with vehicle at P28 as compared to the other three experimental groups (Fig. S1), which reinforces the finding about individual species identified in the specific group comparisons.

### Abundance of *Ruminococcaceae* and other taxa correlate with frontal cortex 5-HT_2A_R density

**Figure 6:**
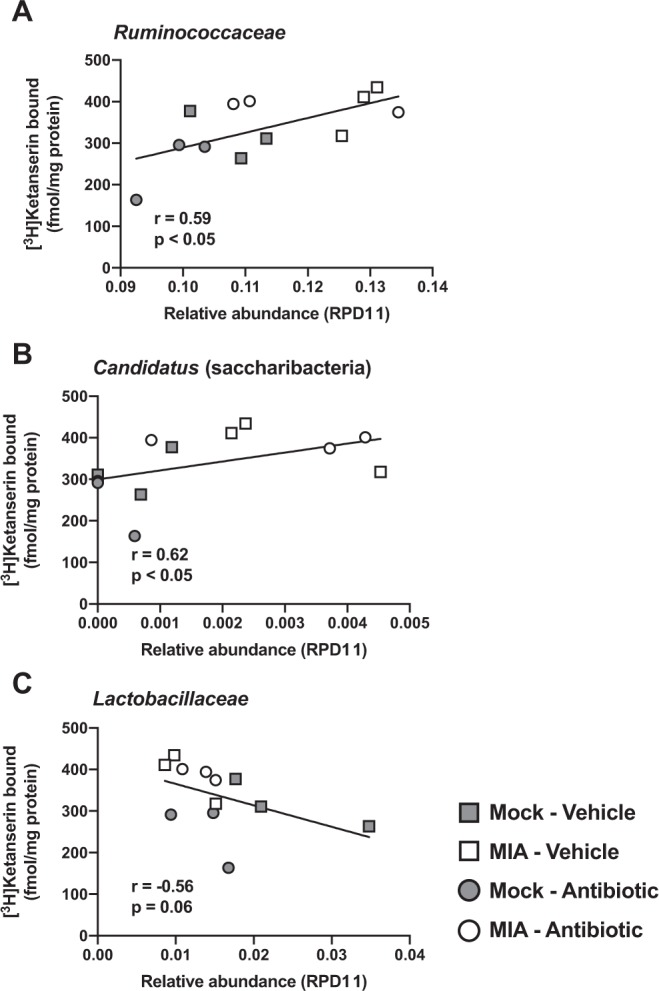
Correlation analysis for the relative abundance of gut bacterial taxa and frontal cortex 5-HT_2A_R density in adult MIA offspring prepubertally treated with antibiotics, and controls. Microbiome abundances are shown as the mean of relative abundance values from the two mice included in each [^3^H]ketanserin binding assay. Correlation analysis was conducted using Spearman’s *r*.

We next assessed whether the alterations in bacterial family in MIA offspring were linked to the density of the 5-HT_2A_R in the frontal cortex. Notably, analysis returned a positive correlation between the relative abundance of *Ruminococcaceae* and frontal cortex 5-HT_2A_R density (Fig. 6A). Relative abundance of *Candidatus* (saccharibacteria) also correlated positively with 5-HT_2A_R density in the mouse frontal cortex (Fig. 6B). Conversely, the trend for a negative correlation between abundance of *Lactobacillaceae* and density of 5-HT_2A_R in the mouse frontal cortex was also evident (Fig. 6C).

## Discussion

Overall, this study investigates the extent to which gut microbiota manipulation at juvenile stages prevents at least part of the negative effects induced by maternal infection with a mouse-adapted influenza virus on behavior models of cognition and expression of the serotonin 5-HT_2A_R in the mouse frontal cortex. Our current data are consonant with the hypothesis that MIA is associated with altered gut microbiota composition in the offspring, and that antibiotic-induced gut microbiota manipulation at juvenile stages prevents the effects of MIA on cognitive deficits later in life. This conclusion is further supported by our microbiota transplantation techniques showing that fecal microbiota transplantation from adult MIA mice to juvenile controls is sufficient to cause behavioral abnormalities during adulthood. Additionally, although antibiotic administration at P28 was unable to reverse the already reported up-regulation of offspring’s frontal cortex 5-HT_2A_R density induced by MIA, we show a very strong correlation between certain bacterial taxa, including *Ruminococcaceae* and *Candidatus* (saccharibacteria) and expression of this serotonin receptor in the mouse frontal cortex.

Previous findings by other groups show that continuous antibiotic depletion of the gut microbiota induces cognitive deficits in mice. Impairment in the novel object recognition test was reported when adult mice (8–11 weeks) received a cocktail of antibiotics via oral gavage for eleven days^50^ or when mice received antibiotic treatment from weaning onwards^51^. The paradigm of gut microbiota manipulation used here is slightly different since we allowed for gut bacterial regrowth following a single antibiotic dose at P28. Accordingly, our findings show a relatively limited effect of prepubertal antibiotic administration in adult mice born to mock-injected mothers, as compared to adult mice born to mock-injected mothers and prepubertally gavaged with vehicle. This absence of negative effects of prepubertal antibiotic treatment is further supported by our behavioral assays. Thus, and opposite to previous findings based on continuous antibiotic administration^50,51^, our results here show that the novel object recognition test was unaffected in adult mice born to mock-infected mothers that had received prepubertal antibiotic treatment, as compared to adult mice born to mock-infected mothers that had received prepubertal vehicle treatment. Nevertheless, certain bacterial taxa were definitely affected by this particular paradigm of prepubertal gut microbiota manipulation. These included enrichment of taxa implicated in host disease, such as *Erysipelotrichaceae*. Additional experimentation will be needed to assess the effects of these bacterial taxa on offspring’s physiology and behavior.

With this in mind, however, an interesting observation in our study is that related to the effect of prepubertal gut microbiota manipulation on MIA-induced cognitive deficits in the adult offspring. As previously shown by a number of experimental MIA models^8,18,21^, maternal infection with a mouse-adapted influenza virus during pregnancy induces deficits in novel object recognition memory. Importantly, a single dose of antibiotic administered via oral gavage at P28 was able to prevent this particular paradigm of deficits in cognitive performance later in life. This behavioral phenotype was observed in parallel with reduction in abundance of components of the gut microbiota that previous work had reported as augmented in fecal samples of patients with schizophrenia, autism and major depressive disorder^14,15,42–44^ – some of these include*, Ruminococcaceae, Porphyromonadaceae, Aerococcaceae*, and *Erysipelotrichaceae*. Still, as above with adult mice born to mock-infected mothers and prepubertally injected with antibiotic, this model of microbiota manipulation at P28 in MIA mice also failed to restore potentially beneficial taxa such as *Lactobacillaceae*. Whether these, in principle, limitations of prepubertal gut microbiota manipulation are compensated by other families that remain unaffected by prepubertal antibiotic administration in the absence of prenatal MIA, such as *Coriobacteriaceae*, or become augmented by prepubertal antibiotic administration in MIA mice, such as *Peptococcaceae*, remains to be investigated.

We also report here that transplantation of gut microbiota from adult MIA mice to prepubertal mock mice is sufficient to induce deficits in recognition memory later in life. This negative effect on novel object recognition is not due to the unwanted effects of the paradigm itself, since it was not observed in mock mice prepubertally receiving gut microbiota transplantation from adult mice born to mock-infected mothers. Our current model of native microbiota “depletion” using a single oral dose of streptomycin was selected based on previous findings by other groups^52^. Although our current results focused on gut microbiota composition and novel object recognition support this experimental design, additional work will consider the inclusion of treatment with a cocktail of non-absorbable antibiotics targeting both Gram-positive and Gram-negative bacteria.

The 5-HT_2A_R is a family A GPCR responsible for the majority of effects of psychedelic drugs, including LSD and psilocybin, as well as involved in the mechanism of action of certain antipsychotic medications, such as clozapine and risperidone^53^. Using the 5-HT_2A_R antagonist [^3^H]ketanserin in radioligand binding saturation curves, we previously reported that 5-HT_2A_R density was increased in postmortem frontal cortex samples (Brodmann area 9) of subjects with antemortem diagnosis of schizophrenia, as compared to control subjects individually matched by gender, age and postmortem delay (time between death and freezing of the samples). Of note, this up-regulation of frontal cortex 5-HT_2A_R density was observed in schizophrenic subjects tested negative for antipsychotic medication in postmortem toxicological analysis, but not in schizophrenic subjects receiving antipsychotic medication^26–28^. Although there are many more genes and pathways that may potentially contribute to the risk of schizophrenia, these findings suggest that this particular serotonergic gene may play a key role in the altered cortical processes of schizophrenia. This concept is further supported by recent GWAS studies in schizophrenia patients and controls^54^.

As discussed above, we previously reported the adult mice born to influenza virus-infected mothers show up-regulation of 5-HT_2A_R in the frontal cortex^29^. A similar phenotype has been observed using a variety of prenatal environmental insults^8,30–32,55^. Here we show that this particular paradigm of prepubertal gut microbiota manipulation did not affect either MIA-induced up-regulation of frontal cortex 5-HT_2A_R or MIA-induced increase in head-twitch behavior upon psychedelic drug administration. In spite of this, a notable finding of the current study is the strong positive correlation of relative abundance of bacterial taxa such as *Ruminococcaceae* and *Candidatus* (saccharibacteria). Frontal cortex 5-HT_2A_R density was, however, negatively correlated with *Lactobacillaceae*. Considering the augmentation of *Ruminococcaceae* previously reported in fecal samples of patients with neurodevelopmental psychiatric conditions^43^, these findings open a new line of investigation focused on the molecular mechanism underlying this connection between certain components of gut microbiota composition and frontal cortex 5-HT_2A_R density.

A still open question is the time period in which environmental factors affect gut microbiota composition. Colonization of the gut microbes after birth is critical to the developing newborn immune system, metabolic function and potentially future health. Nevertheless, our previous observations using a cross-fostering experimental approach showed that certain phenotypes induced by prenatal stress during pregnancy, including DOI-induced head-twitch behavior, were increased in prenatally stressed mice that were raised by unstressed surrogate mothers^8^. A similar concept after cross-fostering has been corroborated by other groups using poly-(I:C)-induced inflammation during pregnancy, also suggesting that maternal gut bacteria during pregnancy promote neurodevelopmental abnormalities in mouse MIA offspring^21^. Recent work also shows that offspring of germ-free (GF) mice “humanized” with fecal microbiota from individuals with autism spectrum disorder displayed behavioral deficits that include repetitive behavior and decreased sociability^56^. We are also aware, however, that maternal perturbations induced by prenatal insults do not stop at birth, but can continue throughout lactation, altering the relationship between pups and dams^57,58^. Further work will be needed to better understand whether MIA-induced maternal perturbations after birth may also affect offspring’s gut microbiota and behavior. In addition, an important limitation of these studies is that they were conducted exclusively in male mice. Future studies including female mice are needed to determine if MIA-induced microbiome and behavioral alterations manifest and respond to antibiotic treatment in a sex-dependent manner.

Metabolomic studies have shown that gut microbial products are found in many extraintestinal tissues, and that molecules derived from the microbiota may influence behavioral phenotypes in mice and humans^59–61^. Some of these include GPCR ligands that mimic signaling molecules^62^. The information we provide here is confined to the potential relation between juvenile antibiotic administration on gut microbiota composition and reduction of MIA-induced behaviors. This information will serve as a basis for future studies to test whether metabolites derived from specific bacterial taxa or species directly and/or indirectly modulate *5-HT*_*2A*_*R* promoter activity or 5-HT_2A_R-dependent function. As a long-term goal, our findings may provide the rationale for the development and testing of microbiome-mediated therapeutic strategies and interventions.

## Materials and Methods

### Animals

Experiments were conducted in accordance with NIH guidelines, and were approved by the Virginia Commonwealth University Animal Care and Use Committee. All efforts were made to minimize animal suffering and the number of animals used. Behavioral testing took place between 9 a.m. and 5 p.m. Animals were housed at 12 h light/dark cycle at 23 °C with food and water ad libitum, except during behavioral testing.

### Cells and viruses

Mouse-adapted influenza virus was propagated in Madin-Darby Canine Kidney (MDCK) cells as previously reported^29^. Viral stocks were stored at –80 °C until viral infections were performed. All experiments with live virus were performed under biosafety level 2 (BSL-2) containment.

### Mouse viral infection

Infection of timed pregnant female mice was performed as previously reported^29^. Briefly, timed pregnant CD1 mice were obtained from Charles River Laboratories. On day 9.5 of pregnancy, mice were anesthetized with ketamine/xylazine before intranasal (i.n.) infection with 5 × 10^3^ plaque-forming units (pfu) of influenza A/WSN/33 (H1H1) virus in 50 µl of PBS. Mock-infected mothers were treated identically but were infused with PBS. Our previous data demonstrate that this sublethal dose of infection causes sickness behavior (lethargy, sleepiness, ruffled fur and lack of grooming), but loss of pregnancy is uncommon^29^. Day 9.5 of pregnancy was chosen because this is neurodevelopmentally equivalent to the end of the first trimester of human pregnancy^63^, a critical period during which environmental insults produce a higher risk of schizophrenia and autism in human offspring. Offspring were separated from their mothers after 3 weeks, and males and females were caged separately in groups of three to five. Subsequent experiments were performed in adult (15–20 weeks) male mice, unless otherwise indicated. Litters were separated according to prenatal manipulation after weaning, and divided over several cages^64^. Three independent cohorts (MIA or mock) of animals were evaluated. Figure 2 includes data from animals in cohorts 1, 2 and 3; Fig. 3 includes data from animals in cohort 2, and Figs. 4–6, Table S1 and Fig. S1 include data from animals in cohort 3. Animals from at least three separate litters were subjected to the different protocols.

### Mouse brain samples

Adult male mice were sacrificed by cervical dislocation, and bilateral frontal cortices (bregma 1.90–1.40 mm) were dissected and frozen at –80 °C for radioligand binding assays. Frontal cortex samples were collected at least one week after the last behavioral assay.

### Radioligand binding

[^3^H]Ketanserin binding assays were performed as previously reported^29^. Briefly, bilateral frontal cortex samples from two mice were pooled together, homogenized using a Teflon-glass grinder, and subjected to the membrane preparation assay. [^3^H]Ketanserin binding (10 nM) was measured at equilibrium in aliquots of frontal cortex membrane preparations that were incubated at 37 °C for 60 min. [^3^H]Ketanserin was obtained from PerkinElmer, and nonspecific binding was determined in the presence of 10 µM methysergide (Tocris). Our previous data show that, under these experimental conditions, specific [^3^H]ketanserin binding is absent in the cortex of 5-HT_2A_R knockout mice^35^.

### Head-twitch behavior

Head-twitch behavior was tested as previously described^35^. Briefly, mice were injected (i.p.) with the hallucinogenic 5-HT_2A_R agonist 1- (2,5-dimethoxy-4-iodophenyl)-2-aminopropane (DOI; 0.5 mg/kg) (Sigma-Aldrich), and 15 min later, they were placed into the center of a Plexiglass cage for 30 min, during which they were videotaped by a video camcorder positioned directly above the cage. Videotapes were scored for head-twitches by an experienced observer blind to prenatal/postnatal manipulations and treatment.

### Novel-object recognition (NOR) test

NOR test was assessed as previously reported^65,66^. Briefly, mice were given a 10-min acquisition trial and a 5-min recognition trial, separated by a 24 h inter-trial return to their home cage. During the acquisition trial, the animals were allowed to explore two different objects (A and B). During the recognition trial, the animals explored a familiar object (A) from the acquisition trial and a novel object (C). Behavior was recorded on video for blind scoring of object exploration. The exploratory preference [100 × (time spent exploring the novel object / total exploration time)] was then calculated for recognition trials.

### Microbiota manipulation

Prepubertal (28 d) mice^67^ born to influenza virus-infected mothers or mock were selected at random for treatment with a single dose of streptomycin (20 mg), or vehicle (distilled water) by oral gavage (200 µl). After this manipulation (Fig. 1), mice were housed with normal drinking water until they became adult animals (15–20 weeks of age).

### Fecal transplantation

For microbiota transplantation, young (28 d) mice received a single dose of streptomycin (20 mg), or mock by oral gavage (200 µl). Three days after this paradigm of antibiotic administration, mice went through a fecal microbiota transfer protocol. Donor mice (two adult mice born to influenza virus-infected mothers, and two adult mice born to mock-infected mothers) were placed in empty autoclaved cages (no bedding) and allowed to defecate normally. The fecal content was resuspended in distilled water (10 ml), and centrifuged for 5 min at 200 ×g. Aliquots of the supernatant were stored at –80 °C for transplantation assays. Inoculation (100 µl of fecal content or distilled water) was performed via oral gavage on two consecutive weeks, for a total of 7 times. After this manipulation, mice were housed with normal drinking water until they became adult animals (10–17 weeks of age).

### 16S rRNA gene sequence analysis

Cecal samples from adult male mice (n = 45 total) were collected and DNA extracted using standard methods, as we have previously reported^68,69^. After the initial filtering of reads by the Ion Torrent software, we selected reads over 250 bp for further processing. We then used the RDP11 Bayesian Classifier to annotate taxa with bootstrap values over 60. Taxa were annotated as unknown if the bootstrap value was below 60. Finally, we removed taxa that were below 0.1% to exclude rare taxa. After this quality filtering, we obtained 1,319,338 high quality reads for further analysis of bacterial composition, with a mean of 31,412.80 reads per sample, ranging from 19,262 to 61,783. Microbiome relative abundances as compositional data were ranged from zero to one. Changes in microbial composition between different experimental groups were assessed using RDP11 (Ribosomal Database Project) Bayesian analysis^70^, QIIME (Quantitative Insights into Microbial Ecology) pipeline^71^, and linear discriminant analysis effect size (LEfSe)^41^. Principal coordinate analysis (PCO) based on Bray-Curtis dissimilarity was performed following standard methods, as we have previously reported^68,69^.

### Statistical analysis

Statistical significance of experiments involving three or more groups across two or more experimental conditions was assessed by two-way or three-way ANOVA followed by Bonferroni’s post hoc test. Statistical significance of relative abundance values was assessed by nonparametric Kruskal-Wallis followed by uncorrected Dunn’s post hoc test. Correlation analysis between [^3^H]ketanserin binding and relative abundance values (mean of relative abundance values from the two mice included in each [^3^H]ketanserin binding assay) was conducted using the Spearman’s *r*. The level of significance was chosen at *p* = 0.05. All data are presented as mean ± standard error of the mean (s.e.m).

## Supplementary information

Supplementary Information - Table S1 and Figure S1.

## Data Availability

Datasets generated during the course of the current study are available, upon reasonable request, from the corresponding author.
