# Supplementary material for: Gut microbiota manipulation during the prepubertal period shapes behavioral abnormalities in a mouse neurodevelopmental disorder model
Source: Sci Rep. 2020 Mar 13;10:4697. doi: 10.1038/s41598-020-61635-6 (PMC7070045; doi:10.1038/s41598-020-61635-6)
Supplement: Supplementary file 1 — Supplementary Information - Table S1 and Figure S1. [file 41598_2020_61635_MOESM1_ESM.pdf]

Supplementary Information

Gut microbiota manipulation during the prepubertal period shapes behavioral abnormalities in a mouse neurodevelopmental disorder model

Justin M. Saunders[1], José L. Moreno[1,*], Daisuke Ibi[1,**], Masoumeh Sikaroodi[4], Dae Joong Kang[2], Raquel Muñoz-Moreno[5], Swati S. Dalmet[4], Adolfo García-Sastre[5,6,7], Patrick M. Gillevet[4], Mikhail G. Dozmorov[3], Jasmohan S. Bajaj[2], and Javier González-Maeso[1]

[1]Department of Physiology and Biophysics, Virginia Commonwealth University School of Medicine Richmond, VA 23298

[2]Division of Gastroenterology, Hepatology and Nutrition, Virginia Commonwealth University and McGuire VA Medical Center, Richmond, VA 23298

[3]Department of Biostatistics, Virginia Commonwealth University School of Medicine, Richmond, VA 23298

[4]Center for Microbiome Analysis, George Mason University, Manassas, VA 20110

[5]Department of Microbiology and Global Health & Emerging Pathogens Institute, [6]Department of Medicine – Division of Infectious Diseases, and [7]The Tisch Cancer Institute, Icahn School of Medicine at Mount Sinai, New York, NY 10029

Supplementary Table S1

Juvenile mice born to influenza virus-infected mothers (MIA) and controls received a single dose of antibiotic via oral gavage, or vehicle. Novel object recognition was tested in adult mice.

	Df	Sum Sq	Mean Sq	F value	*p* value	
**MIA**	1	150	150	4.78	0.03	*
**Antibiotic**	1	32	32	1.03	0.31	
**Task**	1	4267	4267	136.01	2.76×10^{-14}	***
**MIA vs Antibiotic**	1	49	49	1.57	0.21	
**MIA vs Task**	1	16	16	0.49	0.48	
**Antibiotic vs Task**	1	156	156	4.97	0.03	*
**Residuals**	39	1223	31			

P values were obtained based on a three-way ANOVA setup (*$p < 0.05$, **$p < 0.01$, ***$p < 0.001$).

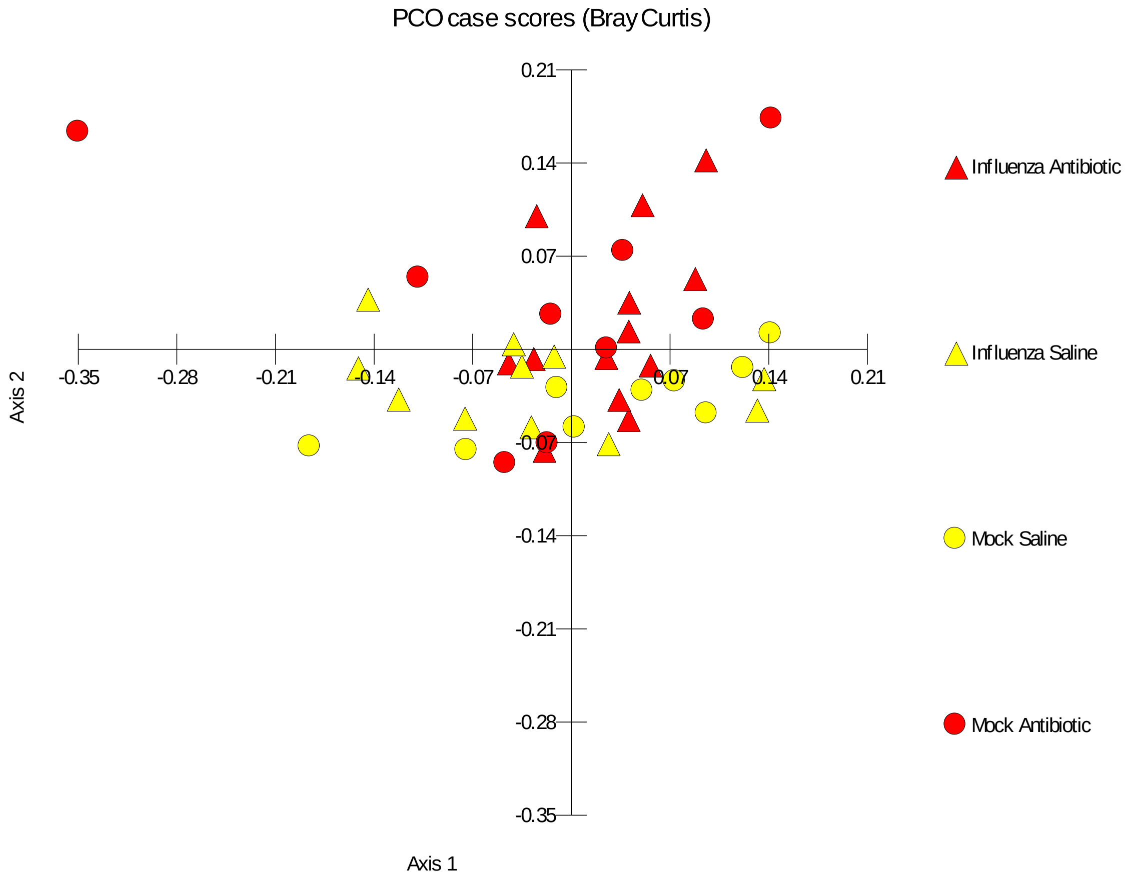

Figure S1. Principle coordinate analysis (PCO) for the gut microbiota from adult offspring of mothers treated with influenza or vehicle during pregnancy. Offspring were treated with either a single dose of antibiotic or vehicle by oral gavage during the prepubertal period.
